# Understanding home delivery in a context of user fee reduction: a cross-sectional mixed methods study in rural Burkina Faso

**DOI:** 10.1186/s12884-015-0764-0

**Published:** 2015-12-11

**Authors:** Manuela De Allegri, Justin Tiendrebéogo, Olaf Müller, Maurice Yé, Albrecht Jahn, Valéry Ridde

**Affiliations:** Institute of Public Health, Medical Faculty, University of Heidelberg, Heidelberg, Germany; Centre de Recherche en Santé de Nouna, Nouna, Burkina Faso; University of Montreal School of Public Health (ESPUM), Montreal, Canada; Institut de recherche en santé publique de l’université de Montréal (IRSPUM), Montreal, Canada

**Keywords:** User fee removal, Home delivery, Mixed-methods study, Burkina Faso, Sub-Saharan Africa, Facility-based delivery

## Abstract

**Background:**

Several African countries have recently reduced/removed user fees for maternal care, producing considerable increases in the utilization of delivery services. Still, across settings, a conspicuous number of women continue to deliver at home. This study explores reasons for home delivery in rural Burkina Faso, where a successful user fee reduction policy is in place since 2007.

**Methods:**

The study took place in the Nouna Health District and adopted a triangulation mixed methods design, combining quantitative and qualitative data collection and analysis methods. The quantitative component relied on use of data from the 2011 round of a panel household survey conducted on 1130 households. We collected data on utilization of delivery services from all women who had experienced a delivery in the previous twelve months and investigated factors associated with home delivery using multivariate logistic regression. The qualitative component relied on a series of open-ended interviews with 55 purposely selected households and 13 village leaders. We analyzed data using a mixture of inductive and deductive coding.

**Results:**

Of the 420 women who reported a delivery, 47 (11 %) had delivered at home. Random effect multivariate logistic regression revealed a clear, albeit not significant trend for women from a lower socio-economic status and living outside an area to deliver at home. Distance to the health facility was found to be positively significantly associated with home delivery. Qualitative findings indicated that women and their households valued facility-based delivery above home delivery, suggesting that cultural factors do not shape the decision where to deliver. Qualitative findings confirmed that geographical access, defined in relation to the condition of the roads and the high transaction costs associated with travel, and the cost-sharing fees still applied at point of use represent two major barriers to access facility-based delivery.

**Conclusions:**

Findings suggest that the current policy in Burkina Faso, as similar policies in the region, should be expanded to remove fees at point of use completely and to incorporate benefits/solutions to support the transport of women in labor to the health facility in due time.

## Background

Assisted delivery by qualified personnel in a health centre is one of the most effective strategies to save the lives of women of childbearing age and helping countries achieve MDG 5 [1, 2]. Although there are many factors determining the use of assisted delivery, and their interactions are complex [3, 4], the financial barriers are very substantial [5]. Consequently, following the recommendations of United Nations agencies and the African Union [6, 7], many African countries have decided to subsidize the cost of deliveries. The objectives of such policies are to increase the proportion of deliveries occurring in health centres and to decrease home deliveries [8]. Surveys show that, while these policies have often been imperfectly implemented, for the most part they have been effective in boosting the use of health facilities [9–11]. Of 11 studies retained in a systematic review looking at the impacts of the delivery subsidy on the use of health facilities surveyed between 1991 and 2011, seven (63 %) demonstrated a positive effect [9].

That being said, despite these gratifying successes linked to eliminating the financial barrier, there are still women in these countries giving birth at home. For example, user fees for normal deliveries were eliminated in Ghana (2005), Burundi (2006), Senegal (2006), and Kenya (2008), and yet 30 % of women still delivered at home in Ghana in 2009 [12], 36 % in Burundi in 2010 [13], 29 % in Senegal in 2011 [14], and 19 % in one district of Kenya at the end of 2011 [15]. Even in Rwanda, presented as a health reform success story [16, 17], 29 % of births in 2010 occurred in women’s homes [18]. For a long time, numerous studies have been undertaken to try to explain why women give birth at home rather than going to a health facility [19].

However, very few studies have examined home delivery in the context of these new delivery subsidy policies. Thus, nearly all the articles retained in a recent systematic review of the determinants of delivery in health facilities had nothing to do with the free care policy [19]. Yet, there have been some studies done in contexts where deliveries are free. In Ghana, one study showed that distance from the health centre, community perception, and awareness of the existence of free care all influenced the use of health facilities [20]. A qualitative study of Ethiopian health workers showed there are many reasons underlying the decision to use health facilities, most notably the lack of resources in health facilities and their failure to satisfy women’s preferences [21]. Another study in the north of that country showed that women who still give birth at home often do so for pragmatic reasons related to their religious beliefs, the influence of older women, the presence of traditional birth attendants, or the ease of remaining at home [22]. In Senegal, distance, absence of means of transportation, and perceptions regarding quality of care are associated with the fact that women still gave birth at home [23]. In Tanzania, deliveries are free, but women have to pay for the required supplies. The use of health facilities in rural areas remains quite limited, notably due to lack of health personnel and of properly equipped health centres, thus many women still give birth at home [24].

Given the scarcity of studies conducted in context of user fee removal/reduction policies, Burkina Faso provides a particularly interesting context in which to study why some women still give birth at home despite the existence of a delivery subsidy policy, which has proven to be very effective. In fact, after banning the use of traditional birth attendants in 2004 [25], in 2007 the government introduced a policy to subsidize normal deliveries in health facilities [25, 26]. This policy was entirely funded by the State’s budget for the period 2007–2015. Indigent women (20 % of the population) are fully exempted from the cost of deliveries (acts and products), while others must pay a fee of 900 F CFA toward the costs incurred for a normal delivery in a primary care health facility. This policy does not include any measures to improve the quality of services provided. According to population-based demographic health surveys (DHS), the rate of assisted deliveries in Burkina Faso rose from 31 % in 1998 to 38 % in 2003, 54 % in 2006, and 66 % in 2010 [27]. The upward trend at the national level intensified after this policy was implemented in 2007 [28]. At the local level, studies that focused on a few districts confirmed significant increases in the use of health facilities and reduction in related household health expenditures [29–32]. Despite this success, the most recent Ministry of Health statistics, whose data quality has been demonstrated [28], showed that 21.7 % of women across the country still gave birth at home in 2011, with significant differences among regions (6.7 % in the capital region vs. 65.1 % in the Sahel region) [33]. Ensuring that each and every woman has access to skilled attendance at birth is of fundamental importance given that birth complications are most often unpredictable and require prompt action by trained personnel. Achieving comprehensive coverage with maternal care interventions represents an essential element of any Universal Health Coverage policy. Furthermore, low rates of maternal mortality and morbidity can be sustained only in settings where all women receive adequate care during labor and birth.

In this article we wish to take advantage of the exceptional circumstances offered by a health and demographic observatory in a rural district of Burkina Faso, to unravel the difficulties that remain in achieving even more comprehensive coverage rates. Similarly to what has been observed in several countries in relation to vaccination coverage [34], Burkina Faso struggles to reach beyond a plateau of 80–85 % for institutional deliveries, with the risk of keeping to exclude those most in need. We pursued our objective by attempting to understand why in the context of a substantial public subsidy some women still give birth at home while others, albeit facing major barriers to access, manage to get to a health facility to give birth.

## Methods

### Study setting

The study took place in the Nouna Health District (NHD), north-western Burkina Faso, in 2011–2012. At the time of the study, the district had a population of approximately 311,000 distributed in 300 villages, and counted 34 first-line facilities, *Centres de Santé et de Promotion Sociale* (CSPS)—33 located in rural areas and one in Nouna town - and one district hospital, also located in Nouna town. The 34 CSPS were equipped and staffed as Basic Emergency Obstetric Care facilities (BEmOC) capable of managing uncomplicated deliveries, while only the district hospital was equipped and staffed as a Comprehensive Emergency Obstetric Care facility (CEmOC) capable of managing complicated deliveries, including C-sections. The government was and continues to be the exclusive provider of formal healthcare services in the area. A sub-portion of the district has been part of a Health and Demographic Surveillance System (HDSS) for over 15 years [35].

### Study design

This study adopted a triangulation mixed methods design [36]. The triangulation design was chosen as the most appropriate mixed methods design since the research question focused on one single phenomenon, i.e. the decision to deliver at home vs. in a health care facility, but aimed at capturing all its possible dimensions, in such a way that neither qualitative nor quantitative methods alone could do [36]. The conceptual framework guiding the study recognized the decision to deliver at home vs. to deliver in a health care facility as the product of the interplay between access factors (acting at the household and at the community level) and individual and household knowledge, beliefs, and practices concerning labor and delivery [37, 38].

The adoption of a mixed methods design allowed us to integrate both series of elements into one single study and to address them from multiple perspectives. The quantitative dimension of the study relied on household survey data to identify socio-demographic, economic, and health system factors associated with the decision to deliver at home. The qualitative dimension of the study relied on a series of in-depth interviews to explore knowledge, beliefs, and practices concerning labor and delivery. Data for the quantitative and the qualitative study components were obtained through two sequential data collection activities which relied on independent data collection tools. Data analysis occurred separately for the two components. At the end, we pooled together quantitative and qualitative findings to derive a final interpretation of the material collected in relation to the over-arching research question.

### Study population, sampling, and data collection

Recognizing that decisions concerning delivery are made collectively [39, 40], the study targeted women with a recent history of delivery, their households, and the community leaders of the villages where these women resided.

#### Quantitative study component

We used data from the 2011 round of a panel household survey conducted in the region since 2006. Data used for this study represents a sub-set of a survey originally designed to monitor progress towards coverage with malaria control interventions and access to maternal care services. Data from previous rounds of the survey has already been used to produce a number of evaluations, including three pertaining to maternal care services [30, 32, 41]. The survey sampling procedures have been described in detail elsewhere [42]. In 2011, data was collected between February and March from a total of 1130 households, selected using a three-stage cluster sampling procedure. First, clusters were defined according to the catchment area of each CSPS (clusters were defined according to the number of CSPS present at baseline, i.e. 27). Second, two villages in each cluster were selected. Third, 20 households were randomly selected in each village, using modified EPI sampling procedures [43]. To take into account its larger population, 70 households were selected in Nouna town.

The survey relied on four core modules to assess a household socio-demographic and economic profile. In the 1130 sampled households, all women who had completed a pregnancy in the twelve months prior to the interview date were administered one additional survey module, gathering information on health care seeking during pregnancy and at time of delivery.

#### Qualitative study component

Based on a preliminary analysis of the quantitative findings, we applied maximum variation sampling to purposely identify the respondents (*n* = 55) for the qualitative study component [44]. We sampled 25 households where women had delivered in a health care facility; 24 households where women had delivered at home; and 6 households which had experienced both home and facility-based deliveries in the prior twelve months. These 55 households were distributed in 13 villages (out of a total of 54 included in the household survey, 24 %), which were purposely selected to display maximum variation in terms of health seeking behavior at delivery. We included villages: i) located at more than 7 km from the health care facility and with less than 80 % of all deliveries taking place in a health care facility; ii) located at more than 7 km from the health care facility and with more than 80 % of all deliveries taking place in a health care facility; iii) located within 7 km of a health care facility and with less than 80 % of all deliveries taking place in a health care facility. We purposely did not include villages located within 7 km of a health care facility and with more than 80 % of all deliveries being facility-based, because we did not see potential to explore remaining barriers to access in such settings. We set the threshold at 7 km because this was the mean distance used to define the CSPS catchment areas on a national level [33]. We set the threshold at 80 % in relation to facility-based delivery because our own data indicated that across the district, 89 % of all women delivered in a health care facility. Within each village, we purposely selected, to the extent possible, households belonging to all socio-economic strata. To do so, we relied on the same quartile classification used for the quantitative analysis. We made the explicit decision to interview both households where women had delivered at home and households where women had delivered in a health facility to be able to explore systematic differences between the two. Our aim in doing so was to understand what made it possible for certain households to overcome barriers which other households were not able to overcome. This decision allowed us to explore possible copying strategies used to overcome remaining barriers to facility-based delivery, in a context of substantial user fee reduction.

In each household, we interviewed the woman having delivered, her husband, and/or any other person indicated by the woman as influential in the process of seeking care at delivery, such as the mother, the mother in law, and/or the household head (in cases where the husband was not the household head). In addition, we interviewed all thirteen village leaders in the selected villages.

All interviews took place in 2012 and were conducted by trained qualitative interviewers, working under the direct supervision of the authors. The interview guide used at the household level was developed to induce respondents to recall the latest completed pregnancy in the household (most frequently the one reported in the household survey) and the decision making process which had led either to a home or to a facility-based delivery. In addition, respondents at the household level were invited to express their opinion on perceived benefits and problems associated with home vs. facility-based delivery, on remaining barriers to access, and on cultural beliefs and practices surrounding labor and delivery. The interview guide used for the village leader did not explore own experiences, but focused exclusively on this latter set of elements. All interviews were conducted in the local languages, tape-recorded, and later *verbatim* transcribed and translated into French.

### Analytical approach

Quantitative household survey data were analysed using Stata 12 (Stata Corporation, Texas, USA). Multivariate logistic regression was used to explore the association between a theoretically relevant set of individual, household head, household, and village characteristics and the outcome variable, defined as “home delivery”. To account for the hierarchical structure of the data, i.e. women are clustered in villages, we applied random effects modelling. In our analysis, we defined women as level 1 and village as level 2. Table 1 provides a comprehensive list of all variables included in the analysis, the answer categories, their distribution in the sample, and the hypothesized coefficient sign. Given the small sample size, we estimated a relatively parsimonious model, including only the most important theoretically relevant and objectively measurable variables identified in prior studies and available in our dataset.

**Table 1:** Variables, their distribution in the study sample, and the expected coefficient sign (Observations (women) = 420; Clusters (villages) = 54)

Variables	Measurement	N	%	Expected coefficient sign
Home delivery		47	11	NA
Woman’s age	0 = ≤ 25 years	214	51	-
	1 = ≥ 26 years	206	49	
Woman’s literacy	0 = Illiterate	366	87	-
	1 = Literate	54	13	
Marital status	0 = Other	308	73	+
	1 = Polygamous marriage	112	27	
History of miscarriage	0 = Had no miscarriage	346	82	+
	1 = Had miscarriage	74	18	
History of ANC attendance	0 = At least 4 ANC visits	145	35	+
	1 = Less than 4 ANC visits	275	65	
Household head’s literacy	0 = Illiterate	270	64	-
	1 = Literate	150	35	
Household socio-economic status (quartiles)	1 = Poorest	NA	NA	-
	4 = Least poor	NA	NA	
Distance to referral CSPS	0 = ≤ 6 km	263	63	+
	1 = ≥ 7 km	157	37	
Location of residence	0 = Within HDSS area	117	28	+
	1 = Outside HDSS area	303	72	

Most variables included in the analysis are self-explanatory. In line with previous research [45–47], socio-economic status was estimated by computing the total monetary value of all animals (cows, sheep, goats, donkeys, horses, pigs, and poultry) and durable assets (cart, plough, telephone, radio, television, bicycle, gas cooker, fridge, and motorbike) owned by the household. The value of each asset was set at the average market price, which was assessed though a parallel small survey carried out at major markets in the district. To compute per capita wealth estimates, we simply divided the total monetary asset value by household size (i.e. number of people living within a household). To align the quantitative and the qualitative analysis, distance to the referral CSPS was computed using 7 km as cut-off point. We included a variable to distinguish households residing in villages under health and demographic surveillance from households residing in villages beyond this area.

Analysis of the qualitative material took place on the transcribed material, directly in French, using a mixture of inductive and deductive coding based on major determinants of access [3, 37]. One of the authors worked as the primary analyst, coding all transcribed material. As a source of triangulation [44], to check the consistency of the emerging interpretation, two senior authors checked the coding scheme, the coding process, and independently read two different sub-sets of the transcripts. We translated into English only the citations used in this manuscript.

The interpretation of the findings as presented in this paper is based on the joint appraisal of the quantitative and qualitative findings. The process of bringing together into major access dimensions [3, 37] findings from the two study components was managed at the end of the two distinguished and parallel analytical approaches.

### Ethical considerations

Institutional ethical review of the study protocol was obtained from University of Heidelberg, Germany and from the Ethical Board of the CRSN, Nouna, Burkina Faso. Oral consent was obtained from all study participants separately for the quantitative survey and the qualitative interviews.

## Results

To simplify reading and highlight the contribution of the single study components, quantitative and qualitative findings are presented separately, but later integrated during the discussion section. Qualitative findings are illustrated through use of direct quotations, extracted from the respondents’ discourse. The respondents’ personal details are omitted, but basic information on their village and their socio-economic status is included next to each quotation to allow the reader to appreciate the variety of backgrounds and experiences included in the sample.

### Quantitative findings

**Fig. 1:**
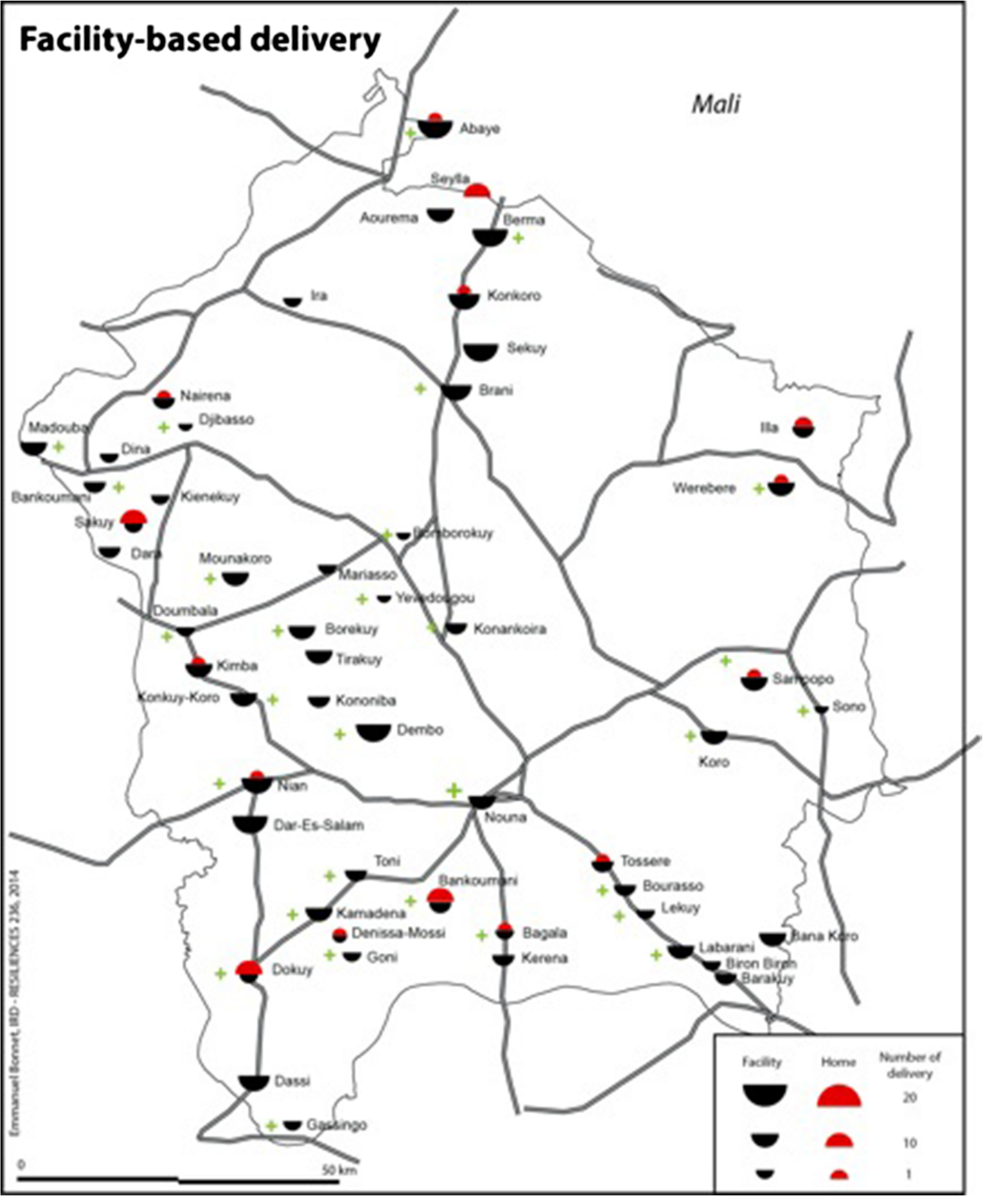
Facility-based and home-based deliveries in the 54 villages included in the household survey of the Nouna Health District. Source: Figure created by Emmanuel Bonnet (geographer) specifically for this article. The authors retain copyright and authorize its distribution open access

In the 1130 households, 420 women reported a pregnancy in the twelve months prior to the survey date. Of those, 47 (11 %, CI 8–14 %) declared having delivered at home. Important differences were observed across the surveyed villages (Fig. 1). In the vast majority of communities (38 villages out of a total of 54 villages), none of the women reporting a pregnancy had delivered at home. In a few selected communities (4 villages out of 54 villages), over 40 % of the women reporting a pregnancy had delivered at home, with a peak of 71 and 100 % in two villages

.

Bivariate analysis identified a positive significant association between home delivery and having attended fewer than four antenatal care (ANC) visits (OR 2.1, CI 1.01–4.35), greater distance to the referral CSPS (OR 12.5, CI 5.44–28.73), and residence outside the HDSS area (OR 3.6, CI 1.39–9.35); while it identified a negative association (although only significant at the 10 % value) between home delivery and a woman’s literacy (OR 1.53, CI.13–1.43) and the highest socio-economic status (OR .36, CI .13–1.01). Random effect multivariate logistic regression only confirmed the positive association between home delivery and greater distance to the referral CSPS (OR 19.33, CI 3.37–110.88) and the negative association with the highest socio-economic status (OR .28, CI .06–1.28) (Table 2). The results of the random effect multivariate logistic regression also indicated a rho of .51 (CI .27–.75), confirmed to be statistically significant from the result of the Likelihood-ratio test of the rho (*p* < 0.001). This suggests the presence of additional village-level variance in the outcome of interest (home delivery), which could not be explained by any of the village-level variables (distance and location of residence) included in the model.

**Table 2:** Unadjusted and adjusted odds ratio for home delivery (Observations (women) = 420; Clusters (villages) = 54)

Variables	Unadjusted estimates		Adjusted estimates	
*Fixed effects*	OR	CI	OR	CI
Woman’s age				
≤ 25 years	1.00		1.00	
≥ 26 years	1.46	.79–2.70	1.42	.59–3.45
Woman’s literacy				
Illiterate	1.00		1.00	
Literate	.43	.13–1.43	.41	.08–2.21
Marital status				
Other	1.00		1.00	
Polygamous marriage	1.06	.54–2.09	.96	.34–2.70
History of miscarriage				
Had no miscarriage	1.00		1.00	
Had miscarriage	1.72	.85–3.50	1.07	.35–3.23
History of ANC attendance				
At least 4 ANC visits	1.00		1.00	
Less than 4 ANC visits	2.10	1.01–4.35	1.44	.49–4.20
Household head’s literacy				
Illiterate	1.00		1.00	
Literate	1.53	.83–2.82	1.19	.46–3.09
Household socio-economic status (quartiles)				
1st quartile	1.00		1.00	
2nd quartile	.84	.37–1.92	.90	.24–3.40
3rd quartile	.88	.39–1.10	.61	.18–2.08
4th quartile	.36	.13–1.01	.28	.06–1.28
Distance to referral CSPS				
≤ 6 km	1.00		1.00	
≥ 7 km	12.50	5.44–28.73	19.33	3.37–110.88
Location of residence				
Within HDSS area	1.00		1.00	
Outside HDSS area	3.60	1.39–9.35	1.69	.26–10.80
*Random effects*				
Intra-cluster correlation coefficient	-	-	.51	.27–.75

### Qualitative findings

All 54 households and 13 village leaders sampled for in-depth interview accepted to participate in the study. All respondents, including the women themselves, their household members, and the village leaders, reported that they understood and valued the benefits of delivering in a health care facility as compared to delivering at home.

*« We prefer to go to the CSPS rather than elsewhere, because at the CSPS, health workers respect pregnant women, they have pity, when they see them suffer. It is because one sees that they do their best, sometimes they go beyond themselves to save certain mothers and their babies.» (Village head; distance to facility > 7 km; village with extremely low rate of home deliveries)*

*« If you deliver in the village, they cannot remove blood from your belly. But at the health facility, they can do it, they can give you drugs and they do an injection. If you deliver at home, you do not recover quickly, let alone be strong enough to work. So, delivering at the health facility is better.» (Household head’s wife; second lowest socio-economic quartile; household with facility-based delivery; distance to facility > 7 km; village with extremely low rate of home deliveries)*

Across villages, women indicated that throughout the course of the ANC consultations, health care providers had insisted on the need to return to the health care facility to ensure a safe delivery. Respondents also consistently indicated that health care providers regularly engage in sensitization activities to ensure that the entire population, and not the women alone, is informed about the importance of delivering in a health care facility. In villagers with high rates of facility-based deliveries, leaders reported having organized additional community gatherings to reinforce the value of an institutional delivery.

*« During my antenatal care visits, health workers gave me a paper which indicated that I should always go back to the facility once labor starts.» (Household head’s wife; second highest socio-economic quartile; household with facility-based delivery; distance to facility > 7 km; village with low rate of home deliveries)*

*« The health workers have conducted an extensive sensitization campaign. It is because of this that we go to deliver at the health facility.» (Household head’s son; second highest socio-economic quartile; household with facility-based delivery; distance to facility > 7 km; village with extremely high rate of home deliveries)*

*« We held a village gathering (…) we explained to people that it is necessary to take their women to the health facility as soon as their women enter labor.» (Village leader; distance to facility < 7 km; village with extremely low rate of home deliveries)*

A few respondents reported that to encourage women to deliver in a CSPS, providers impose both a fine for home delivery (ranging from CFA 3000 to CFA 5000) and the payment of high fees to women who seek postnatal care, but who have previously delivered at home. Respondents explained that fear to have to pay such high fees indirectly creates an obligation for all women to deliver in a health care facility. In a few selected instances, fear to face the fine discouraged women who had delivered at home from seeking further care for themselves and their newborns.

*« When we went to the health facility after having delivered at home, health workers were not happy. They reproached us, saying that women should not deliver at home. But they did check me and my child, although they made us pay a 5000 CFA fine for home delivery. » (Recent parturient; second highest socio-economic quartile; household with home delivery; village hosting CSPS; village with low rate of home deliveries)*

*« I did not go to the CSPS with my child even after the delivery (…) because I was afraid that they would make me pay a fine. There are regulations against home delivery.» (Recent parturient; lowest socio-economic quartile; household with home delivery; distance to facility > 7 km; village with low rate of home deliveries)*

The vast majority of village leaders explicitly referred to the user fee reduction policy introduced in 2007 as the reason motivating the substantial reduction in the price for facility-based delivery experienced over the last few years. Household respondents instead, did not mention the SONU policy. Still, they were perfectly aware that an uncomplicated delivery should be charged 900 CFA, but knew that in practice real costs often amount to a total of 1500 CFA.

Across socio-economic strata, households did not indicate the price for facility-based delivery as the decisive element discouraging them from delivering in a CSPS. Respondents, however, generally reported a need to prepare during the months of the pregnancy to be able to meet the cost of delivering in a health facility. In villages with higher rates of facility-based delivery, respondents explicitly mentioned pooling resources across community members to meet the cost of a facility-based delivery, in case of need.

*« We do not have financial problems, not of the kind that could justify not going to the facility and being unable to pay the fees.» (Recent parturient; second highest socio-economic quartile; household with home delivery; village hosting CSPS; village with low rate of home deliveries)*

*« In our village, when a woman is pregnant, people usually save money to be able to pay for the delivery fees.» (Recent parturient; second highest socio-economic quartile; household with home delivery; distance to facility > 7 km; village with low rate of home deliveries)*

*« In our village, all women and all men belong to a cooperative. During the rainy season, everyone works collectively on the fields as part of such cooperative. This collective work brings revenues which are stored in the cooperative treasury. In case a member, whether woman or a man, does not have the financial means to pay for healthcare, he or she can ask the cooperative for a loan and the cooperative grants it to him or her to solve the problem.» (Village leader; distance to facility > 7 km; village with low rate of home deliveries)*

Geographical accessibility was unanimously identified as the most prominent barrier to reaching a health facility for delivery, across villages with high and with low rates of facility-based delivery. Geographical accessibility was not described in terms of mere distance to the health facility, but more comprehensively in relation to the state of the roads, especially during the rainy season, and the prompt availability of adequate means of transport when labor starts.

*« It is the same problem (the geographical accessibility) during both seasons. But, during the rainy season, you can reach the river and you find that the water is s high that you must wait 24 h before the water gets lower again and you can cross the river. The road is not good: there are stones, wholes, sand. You cannot transport a woman in labor on a bike; even with a motorbike, it is difficult once labor has started. We recommend taking a cart, which serves as our ambulance.» (Household head; second highest socio-economic quartile; household with facility-based delivery; distance to facility > 7 km; village with extremely high rate of home deliveries)*

However, none of the households where women had delivered at home attributed the decision directly to the bad state of the roads. These households rather insisted on the short length of the labor, not leaving enough time to organize adequate transport for the parturient. In contrast, households where women had delivered in the health facility appeared to be better prepared for the time of labor, having pre-arranged transport in advance. In addition, households residing in villages distant from the referral facility, but with high rates of facility-based delivery indicated a certain level of collective organization and solidarity to organize transport to the facility.

*« She entered labor very early in the morning. We went out looking for a motorbike. By the time we returned home, she had already delivered. » (Household head’s daughter in law; highest socio-economic quartile; household with home delivery; distance to facility > 7 km; village with high rate of home deliveries)*

*« We transport the woman in labor on a motorbike, sitting in the middle between two other people. If your wife is pregnant and you do not have your own motorbike, you ask someone else in the village to lend you one.» (Household head; highest socio-economic quartile; household with facility-based delivery; distance to facility > 7 km; village with extremely low rate of home deliveries)*

*« I approach personally all households where I know that there are expecting mothers to ask whether they attend antenatal care. If I find out that this is not the case, I sensitize them (to the importance of the issue) and follow them up. When someone’s wife enters labor, he comes to see him. I tell him to look for a motorbike, so we can take his wife to the CSPS.» (Village leader; distance to facility > 7 km; village with extremely low rate of home deliveries)*

Delays in organizing transports were often associated with the absence of the household head at the time of labor. Household heads were in fact unanimously recognized as the sole being able to authorize and organize transport for the women in their households. In communities with high rates of facility-based deliveries, however, women reported that, notwithstanding the authority of the household head, the collective expectation that women should deliver in a health facility had recently empowered them to make the decision autonomously, if needed.

*« The time of her delivery coincided with the time of the year when we are working full-time in the fields. This is why I could not be home to take her to the facility.» (Household head; highest socio-economic quartile; household with home delivery; distance to facility > 7 km; village with extremely high rate of home deliveries)*

*« It is the husband who decides. But if he is not there, we (women) can decide on our own to go deliver in a CSPS, because these days everyone delivers in a CSPS.» (Household head’s wife; highest socio-economic quartile; household with facility-based delivery; distance to facility > 7 km; village with extremely low rate of home deliveries)*

Only in three villages, households attributed their own decision and the decision of others in the community to deliver at home to socio-cultural factors. In one village with a rate of home deliveries just above 40 %, preference for home delivery above facility based delivery was related to a recent case of maternal death having occurred at the facility, leaving the community to speculate on the causes of such death. In two other villages (with rates of home deliveries of 71 and 100 % respectively), the decision to deliver at home was motivated by the rooted belief that delivery should occur in one’s own village. Facility-based deliveries were therefore systematically avoided not out of fear of delivering institutionally, but out of a wish not to leave one’s own village during labor and delivery.

*« When a woman needs to deliver, she delivers in our village. If she delivers outside the village, she becomes crazy.» (Village leader; distance to facility < 7 km; village with extremely high rate of home deliveries)*

*« The belief in our village is that no woman should deliver outside the village. It is due to our customs that we refuse to go there (to a CSPS located in another village).» (Household head; distance to facility < 7 km; highest socio-economic quartile; household with home delivery; village with extremely high rate of home deliveries)*

## Discussion

### Acting on all barriers to access to assisted deliveries

The objective of this article is not to evaluate the effectiveness of the delivery subsidy policy that has been implemented since 2007 in Burkina Faso, since the topic has already been covered extensively in several other articles [30, 32, 48]. In the case of this study, the policy represents the key contextual factor and the starting point to understand why, in spite of the presence of an explicit effort to drastically reduce fees at point of use, many women still deliver at home while others manage to overcome existing barriers to deliver in a health facility. As stated in the introduction, our aim was to understand what difficulties remain to achieve even more comprehensive coverage rates, ensuring effective coverage for all concerned women. Effective coverage of all women represents an essential element of any Universal Health Coverage policy and it is key to achieving sustained and equitable reductions in maternal mortality and morbidity.

In the district where we carried out the study, 90 % of women deliver in a health facility with qualified personnel. This is an impressive record for the West African region [9, 12], and the subsidy policy has been a major contributor to this success. Still, our study suggests that many other actions, beyond the mere reduction of user fees, are likely to have contributed to increase the number of facility-based deliveries. In our study, health workers, often cited for poor behaviour [49], are consistently reported to have promoted facility-based delivery. Thus, we could define them as political entrepreneurs [50], actively encouraging women to make use of the new policy and give birth in health facilities. This finding is suggestive that relationships between health workers and populations, as well as people’s perceptions of quality of care, are important factors in the use of assisted deliveries [3, 51, 52]. A recent study in a northern district of Burkina Faso that compared CSPSs with different levels of facility-based deliveries confirmed the importance of the provider-woman relationship in shaping decisions regarding delivery [48]. Our study, however, also indicates that it is not only provider-woman relationships that matter, but also those with the entire community. Our qualitative findings clearly indicated that the support that a community could show women, by facilitating transportation during labor or by helping to cover the costs associated with the delivery, played a key role in enabling women to get to the health facility in due time.

Our study also suggests that citing cultural factors, as it is often done in some discourses, as a central factor explaining home deliveries is not justified [53]. We found no strong indication to support this argument, aside from a few exceptional situations, which were linked to the location of the health centre and not even to an explicit wish to avoid contact with Western care during labor and delivery. Further support in favor of this argument comes from the fact that even women who delivered at home, actually valued giving birth in a health centre and would have done so had they had a health centre in their own community.

Our study indicates that for this type of analysis concerning policies and health systems, an understanding of context and social relation is essential [54], even though it appears that very few studies to date have applied such an approach to explore this matter systematically in Africa [9].

### Still some challenges to overcome so that all women can deliver with qualified personnel

In addition to shading light on the contextual and social elements that contributed to the high uptake of facility-based delivery in Burkina Faso, this study suggests that several barriers to access persist, so that many women continue to give birth at home. The data presented in this article highlights the multiplicity of determinants underlying the decision to deliver in a health centre [3] and calls for further studies to provide additional evidence on the matter.

Although point-of-service financial barriers were not directly mentioned by households during the qualitative interviews, the quantitative data identified a clear trend (albeit not significant) suggesting that poorer households were more likely than wealthier ones to have experienced a home delivery. This finding is not surprising and well aligned with the broad literature on health care seeking [1, 9, 11]. To this regard, the interviews illuminated the understanding of the quantitative findings as they revealed that households’ difficulties to meet the costs associated with the entire care process, rather than just the official fees alone. One ought to consider that households’ financial capacities remain very limited in this context of generalized poverty in this isolated rural setting. Thus, the promise made by Burkina Faso’s President in 2010 to abolish fees for delivery completely, an important step still waiting to be organized [55], could potentially further reduce home deliveries in the study area.

This promise is all the more important, given that it was endorsed by the African Union [6] and is featured in the national social protection policy adopted in 2012 [56]. Increasing public funding and eliminating point-of-service user fees for deliveries also has an impact on women’s autonomy. In fact, in this context, women must not only ask their husband for permission to go to the health centre, but also for financial resources to pay for care [57]. Eliminating user fees can therefore also have an impact on women’s empowerment and their ability to get to a health centre, as was shown by preliminary in Burkina Faso [58] and confirmed by the statement of a few women in selected villages represented in this study.

In line with evidence from Senegal and with the overall evidence on home delivery [23, 38, 40], both the quantitative and the qualitative findings clearly pointed at the fact that geographical access remains the single most important barrier for women to deliver in a health facility. The qualitative findings explained how the large positive association between home delivery and distance to the referral facility detected in the multivariate analysis actually plays the most important role in shaping households’ decisions at labor. Households described geographical barriers not in terms of mere distance, but in relation to the poor state of the roads and the lack of adequate transportation means. In turn, these two elements translate in high transaction costs, which are much more important than the mere fees medical fees in determining households’ decisions. In fact, the national delivery subsidy policy provides free transportation from the health centre to the district hospital for obstetric emergencies [26], but no provision has been made for transportation from the village to the health centre. Innovative solutions, such as maternity waiting homes or transportation vouchers [5], which need to be organized, must certainly be the next urgent priority for the State if it intends to achieve universal delivery coverage for its women [51]. In the case of very isolated villages, it may also be useful to test the option of installing a qualified health worker, as Burkina Faso appears to have many health workers comparing to other countries in the region [59], to assist deliveries directly in the village. This may prove to be a successful strategy, since our study clearly indicates that, while women may not want to give birth at home, they are often simply obliged by circumstances to do so.

Both the quantitative (by detecting village-level variance) and the qualitative findings suggested the existence of within-district inequalities due to the fact that, in the absence of any national solution, each community does what it can to enable facility-based delivery with the means at its disposal. In line with prior research [32, 47], probably due to wider exposure to health messages and access to resources beyond the ones disposed by the State, multivariate analysis identified a non-significant yet remarkable trend for communities in the HDSS area to experience lower levels of home deliveries than communities in the non-HDSS area. The qualitative findings complemented this quantitative observation, clearly pointing at how communities displayed different levels of collective organization around women’s delivery needs. So, while some communities imposed fees to refrain women from delivering at home, others mobilized existing social networks (such as agricultural cooperatives) to pool funds to cover the costs associated with a facility-based delivery.

In a context of administrative decentralization, it is often surprising that local solutions are suppressed on the pretext of maintaining implementation fidelity at the national level. With regard to access to CSPSs in several health districts, some rural communities decided to levy a tax on households in which women still gave birth at home [48]. Another district decided to go beyond the 80 % subsidy provided by the State and to use their own public resources to completely eliminate user fees for women [60]. However, in all cases, these initiatives were stopped by the central level, which wanted all districts to respect the content of the national policy. While theory tells us that a policy’s implementation fidelity sometimes guarantees its effectiveness [61], for public health interventions to succeed it may be essential to allow local actors to make adaptations geared to the local context and to employ innovative strategies [62]. A similar situation was seen in neighbouring Niger where, because of failures in the national caesarean subsidy policy, local actors instituted a local taxation system that allowed ambulances to continue functioning. The central State wanted this system shut down, as it wanted the user fees exemption to be respected in accordance with its policy—even though it was not reimbursing the health centres, thereby rendering the policy ineffectual [17].

### Methodological considerations

The obvious strength of this study lays in the joint use of quantitative and qualitative methods of data collection and analysis, allowing us to explore a multitude of factors which the single methodologies would have not been able to capture adequately. Specifically, the possibility to draw the qualitative sample from the same pool of respondents as the quantitative sample and on the basis of purposive criteria identified through preliminary quantitative analysis increases the credibility of the study by allowing for greater comparability across data collection methods. Similarly, having interviewed several household members, rather than women alone, as well village leaders increased our capacity to triangulate information across data sources, adding to the overall validity of the study.

Still, we ought to acknowledge a number of important methodological limitations. First, the sample size for the quantitative analysis was relatively small (albeit aligned with the sample size from previous survey years [30, 32]), possibly explaining why significance levels could be achieved only for a limited number of explanatory variables. The power to achieve statistically significant results was further hampered by the application of hierarchical modelling. The relatively small sample size also explains why we opted for a two-level model (woman and village) rather than for a three-level model (woman, village, and health facility), although the latter would have been conceptually preferable. Furthermore, we acknowledge that the model could have benefitted from adding information pertaining to the quality of the services on offer at the various CSPS. The facilities are comparable in relation to equipment and staffing, but we cannot exclude differences in quality of care linked to the leadership of each facility. Such information, however, cannot be collected retrospectively and was not available for the year of our quantitative survey (2011). Thus, it could not be included in the model. Similarly, additional information on the characteristics of the villages would have increased the model’s explanatory power and possibly reduced the size of the rho. Such information, however, was not available to us. Second, the time which elapsed between the quantitative and the qualitative data collection rounds might have made it difficult for the respondents to recall with precision the decision-making process surrounding the event of the prior delivery. We can neither exclude recall bias nor the possibility that the respondents’ attitudes concerning home vs. facility-based delivery had changed during this period of time. Third, we cannot exclude that having interviewed women in their households, rather than in a neutral setting, might have made it more difficult for them to describe as freely as they would have wished the decision-making process surrounding their delivery. We can therefore not exclude that in some instances, respondents were inclined to provide socially acceptable answers, to please not only the interviewer, but the other household members. Last but surely not least, we need to acknowledge the limited generalizability of the findings beyond the Bukinabè context, given that the study was conducted in a context where the partial removal of user fee had induced an increase in service utilization not observed elsewhere in SSA. Nevertheless, we trust that the thick description of the context will allow the reader to judge if and to what extent, the policy implications that follow from our findings also apply to other contexts. We trust that more studies like ours be conducted in the future to evaluate not only the impact of interventions, but the contextual factors affecting their implementation and their success or failure [48, 63]. Such studies are needed to confirm the initial suggestive evidence emerging from our study.

## Conclusions

Beyond the achievements of the user fee reduction policy already reported in previous studies, our study points at the existence of remaining barriers to access delivery services, urgently calling for further policy intervention. It is desirable that the former President’s promise to fully abolish fees at point of use for maternal care services is translated into actual practice as soon as possible. The opportunity to revise the current policy for the better comes from the fact that provisions for its implementation, including budgetary commitment, are only set up to 2015. Therefore, this year represents an important turning point, where budgetary commitment needs to be renewed, but where content can also be revised to remove the current cost-sharing and to introduce additional benefits, such as coverage for transport costs for all women in labor. Such a development would be most beneficial in the light of the international discourse surrounding the post-MDG agenda, which clearly recognizes the need for further action to reduce maternal and neonatal mortality, and given the recent public statement by the World Bank in disfavor of user fees in sub-Saharan Africa.
